# Differential Effects of Combination of Renin-Angiotensin-Aldosterone System Inhibitors on Central Aortic Blood Pressure: A Cross-Sectional Observational Study in Hypertensive Outpatients

**DOI:** 10.1155/2020/4349612

**Published:** 2020-09-07

**Authors:** Akshyaya Pradhan, Pravesh Vishwakarma, Monika Bhandari, Rishi Sethi, Varun Shankar Narain

**Affiliations:** Department of Cardiology, King George Medical University, Shah Mina Road, Chowk, 226003, Lucknow, Uttar Pradesh, India

## Abstract

**Background:**

Central aortic blood pressure (CABP) indices, central hemodynamics, and arterial stiffness are better predictors of cardiovascular events as compared with brachial cuff pressure measurements alone. The present study is aimed at assessing the effects of different antihypertensive drug combination regimens involving renin-angiotensin-aldosterone system (RAAS) inhibitors on CABP indices in Indian patients with hypertension.

**Methods:**

This was a cross-sectional, single-center study conducted in patients treated for hypertension for >6 weeks using different treatment regimens involving the combination of RAAS inhibitors with drugs from other classes. CABP indices, vascular age, arterial stiffness, and central hemodynamics were measured in patients using the noninvasive Agedio B900 device (IEM, Stolberg, Germany) and compared between different treatment regimens.

**Results:**

A total of 199 patients with a mean age of 54.22 ± 10.15 years were enrolled, where 68.8% had hypertension for over three years and 50.25% had their systolic blood pressure (SBP) < 140 mmHg. Combination treatment with angiotensin II receptor blockers (ARBs) and angiotensin-converting enzyme inhibitors (ACEIs) was given to 77.9% and to 20.1% patients, respectively. The mean vascular age was higher than the actual age (58.13 ± 12.43 vs. 54.22 ± 10.15, *p* = 0.001). The SBP and diastolic blood pressure (DBP) levels in patients treated with ACEI-based combinations were lower than those in patients treated with ARB-based combinations (*p* < 0.05). The mean central pulse pressure amplification, augmentation pressure, and augmentation index were lower in patients treated with ACEI-based combinations than those treated with other treatments (*p* = 0.001). In a subgroup analysis, patients given perindopril and calcium channel blockers (CCBs) or diuretics had significantly lower CABP and pulse wave velocity than those given other treatments (*p* < 0.05). A total of 6.5% patients experienced any side effects.

**Conclusion:**

The majority of central hemodynamic parameters, including vascular age, were found to improve more effectively in patients treated with ACEIs than with ARBs. Our results indicate a gap between routine clinical practice and evidence-based guidelines in Indian settings and identify a need to reevaluate the current antihypertensive prescription strategy.

## 1. Introduction

Hypertension is one of the most common causes of premature death worldwide according to the World Health Organization [1]. Globally, hypertension is estimated to afflict nearly one billion people, accounting for 26% of the population, and is a primary modifiable risk factor for stroke and heart disease, which are among the top leading causes of deaths worldwide [2]. The overall burden of hypertension in India is 29.8%, which translates to 33.8% prevalence in urban population and 27.6% in rural population [3]; therefore, it continues to be a major public health challenge.

Conventionally, hypertension is managed on the basis of brachial blood pressure (BP).

Nevertheless, emerging evidence shows that central aortic blood pressure (CABP) predicts cardiovascular events more effectively than brachial blood pressure [4]. In addition to conventional BP measurements, other variables such as advanced hemodynamic parameters, including stroke volume, cardiac output, peripheral resistance, arterial stiffness (as measured by pulse wave velocity), and vascular age, are also analyzed to obtain critical information about cardiovascular health [5, 6]. Moreover, studies have shown that the predictive value of CABP is independent of the corresponding brachial blood pressure [7–10]. Although CABP has been demonstrated to be a valuable predictor of cardiovascular outcomes, its use in the routine clinical practice is very confined, which limits its prognostic utility.

Renin-angiotensin-aldosterone system (RAAS) inhibitors, calcium channel blockers (CCBs), diuretics, and *β*-blockers are the commonly recommended antihypertensive drugs. These drugs have different modes of action, which are conventionally studied with respect to peripheral BP. However, evidence shows that despite the similar reduction in the peripheral BP, they have differential effects on the central BP [11–13]. These effects have been studied extensively globally; the data originating from India are scarce owing to the limited use of CABP as a prognostic tool in routine practice. Technological advances have led to the development of various noninvasive devices to estimate central BP, which renders these parameters amenable to a multitude of patient populations and disease states [14–17]. Using the cuff-based Agedio B900 (monitor PWA) device integrated with Agedio K500, we evaluated the effects of various antihypertensive drugs on CABP indices in hypertensive outpatients in routine clinical practice in India.

## 2. Materials and Methods

### 2.1. Study Design and Participants

This was a cross-sectional observational study conducted at a Tertiary Care Medical University, Lucknow, from January 2017 to December 2018. A total of 240 patients were screened; of which, 199 patients who were on stable antihypertensive dose for over six weeks or who were previously or recently treated with antihypertensive medications were included in the study. Target BP control was defined as brachial SBP < 140 mmHg or DBP < 90 mmHg. Patients with liver dysfunction, signs and symptoms of heart failure, chronic kidney disease (CKD), or systemic inflammation and infection were excluded from the study.

The study was approved by the institutional ethics committee. Written informed consent was obtained from all patients.

### 2.2. Study Size

Eligible patients coming to the outpatient department (OPD) during the defined time period were taken for the study. The approximate number according to previous medical records is around 180-200 patients.

### 2.3. Measurement of Hemodynamic Parameters

The noninvasive Agedio B900 device (IEM, Stolberg, Germany), which works based on systolic pressure amplification phenomenon, was used to measure peripheral BP, central BP, advanced hemodynamic indices, arterial stiffness, and vascular age [18–21]. The various indices measured were as follows: peripheral and central systolic BP (SBP) and diastolic BP (DBP), pulse pressure (PP), mean arterial pressure (MAP), PP amplification (PPA), age of vessels (older than biological age or same as the biological age, years), peripheral resistance (PR), cardiac output (CO), stroke volume (SV), cardiac index (CI), augmentation pressure (AP), augmentation index (AI), reflection coefficient (RC), and pulse wave velocity (PWV). These indices were compared in patients receiving different antihypertensive drug combinations. In all patients, BP and PWV were measured in an ideal environment (sitting and quiet position). A subgroup analysis was also carried out to compare the CABP indices in patients who were on the ACEI perindopril with the rest of the patients.

### 2.4. Statistical Analysis

Descriptive statistics was performed on all demographic and clinical measurements. Baseline patient characteristics were reported as percentages for categorical variables and mean ± standard deviation for continuous variables. Data comparing means of three or more groups were analyzed using the two-way analysis of variance (ANOVA) test (SPSS Inc., Chicago, USA), and data involving comparison of means between only two groups were analyzed using *t*-test. Post hoc analysis of various groups analyzed by ANOVA test was also performed. All *p* values less than 0.05 were considered significant.

## 3. Results

### 3.1. Demographic and Clinical Characteristics

A total of 199 patients were analyzed in the study; out of them 59.3% were men. The age of patients ranged from 27 to 78 years, with the mean age (±SD) being 54.2 ± 10.15 years. Only 36.2% patients had controlled hypertension. A majority of the patients (67.8%) had hypertension for or over three years. The family history of hypertension was seen in 69.3% patients, 18.6% were smokers, 15.1% chewed tobacco, and 5.5% had diabetes (Table 1). A total of 13 (6.5%) patients experienced side effects, three cases of cough, four cases of edema, and six patients reported other side effects.

All the 199 patients were on antihypertensive medication; 45.2% were on angiotensin II receptor blocker (ARB) and diuretics (ARB+diuretics), 32.7% were on ARB and CCB (ARB+CCB), 13.6% were on angiotensin-converting enzyme inhibitor (ACEI) and diuretics (ACEI+diuretics), and 6.5% were on ACEI and CCB (ACEI+CCB), while 2% were on other drugs (Table 2). Subgroup analyses revealed that a total of 22 patients were on the long-acting ACEI, perindopril.

### 3.2. Peripheral Blood Pressure Measurements

**Figure 1:**
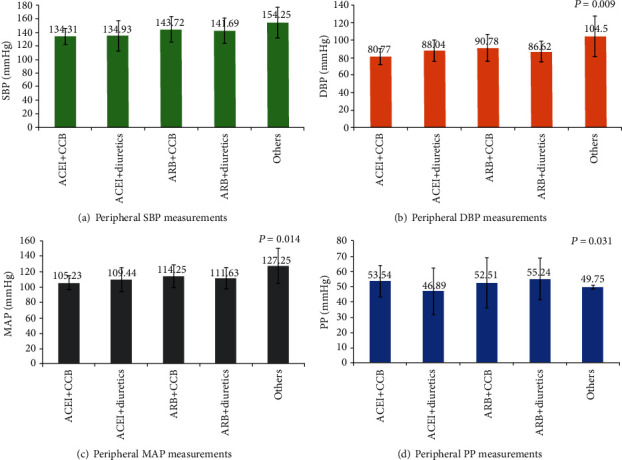
Peripheral blood pressure measurements among various antihypertensive drug combinations. SBP: systolic blood pressure; DBP: diastolic blood pressure; MAP: mean arterial pressure; PP: pulse pressure.

Overall, 100 patients had their SBP < 140 mmHg and 109 patients had their DBP < 90 mmHg. As shown in Table 3 and Figure 1, the mean peripheral SBP and DBP were significantly different among the treatment groups (*p* = 0.027). The mean SBP was the lowest in patients receiving ACEI+CCB (134.31 ± 11.35 mmHg), followed by those in the ACEI+diuretics (134.93 ± 21.96 mmHg), ARB+diuretics (141.69 ± 18.49 mmHg), ARB+CCB (143.72 ± 18.62 mmHg), and ARB+diuretics (141.69 ± 18.49 mmHg) groups. The mean peripheral DBP was 90.78 mmHg in the ARB+CCB patient group, which was significantly higher (*p* = 0.009) than 80.77 mmHg in the ACEI+CCB group. The peripheral DBP between other groups was comparable. The mean peripheral MAP was 114.25 mmHg in the ARB+CCB group, which was significantly higher (*p* = 0.014) than 105.23 mmHg in the ACEI+CCB group; other treatment groups had comparable mean peripheral MAP. The mean peripheral PP was 55.24 mmHg in the ARB+diuretics group, which was significantly higher (*p* = 0.031) than 46.89 mmHg in the ACEI+diuretics group, while that of others was comparable (Table 3). Overall, all the peripheral BP measurements were noted to have improved in patients on ACEIs compared with those on ARBs.

### 3.3. Central Blood Pressure Measurements

**Figure 2:**
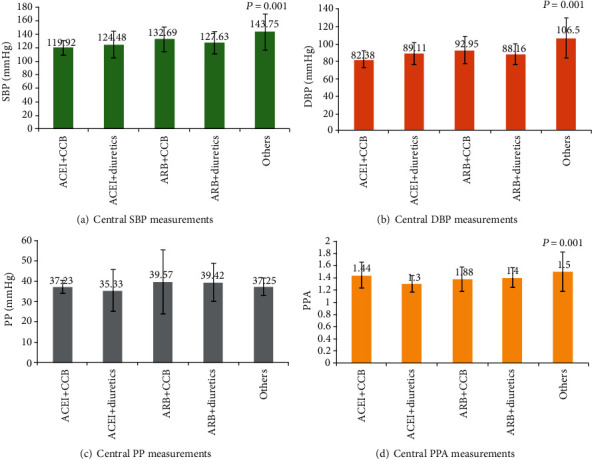
Central blood pressure measurements among various antihypertensive drug combinations. SBP: systolic blood pressure; DBP: diastolic blood pressure; PP: pulse pressure; PPA: pulse pressure augmentation.

The mean central SBP in the ACEI+CCB group (119.92 ± 10.39 mmHg) was significantly lower than that in the ARB+CCB group (132.69 ± 18.26 mmHg) (*p* = 0.001); central SBP in others was comparable. A similar pattern was observed for the mean central DBP (ACE+CCB: 82.38 ± 09.33 mmHg vs. ARB+CCB: 92.95 ± 15.33 mmHg (*p* = 0.001)). Although the mean central PP was higher in the ARB+CCB (39.57 ± 15.53 mmHg) and ARB+diuretics (39.42 ± 09.31 mmHg) groups than in the ACEI+CCB (37.23 ± 03.11 mmHg) and ACEI+diuretics (35.33 ± 10.12 mmHg) groups, these differences were not significant (*p* > 0.05). The mean central PPA was 1.44 ± 0.21 in the ACEI+CCB group and 1.40 ± 0.16 in the ARB+diuretics group, which was significantly higher (*p* = 0.001) than 1.30 ± 0.13 in the ACEI+diuretics group. The mean HR was 92.63 ± 14.12 beats/min in the ARB+CCB group, which was significantly higher (*p* = 0.006) than 68.56 ± 05.52 beats/min in the ACEI+diuretics group and 78.11 ± 12.47 beats/min in the ARB+diuretics group (Table 4, Figure 2). Similar to the peripheral BP readings, the central BP levels were higher in patients who were on ARB than those on ACEI.

### 3.4. Vascular Age

In the overall cohort, the mean vascular age of patients was significantly higher than the average actual age (58.13 ± 12.43 years vs. 54.22 ± 10.15 years (*p* = 0.001)). The mean vascular age was the lowest in the patients in the ACEI+diuretics treatment group (53.30 ± 13.38 years). The mean vascular age was 60.55 ± 10.77 years in the ARB+CCB group and 60.85 ± 09.25 years in the ACEI+CCB group, which was significantly higher (*p* = 0.034) than 53.30 ± 13.38 years in the ACEI+diuretics group and for others groups was comparable (Table 5).

### 3.5. Arterial Stiffness

The measurement of arterial stiffness via PWV, AP, and AC gives an assessment of the vascular age. The mean AP was significantly lower in the ACEI+diuretics group (7.11 ± 04.67 mmHg) than 11.97 ± 09.71 mmHg in the ARB+CCB group (*p* = 0.001), and it was comparable between other treatment groups. The mean AI was 17.67% ± 12.16% in the ACEI+diuretics group, which was significantly lower than 34.82% ± 15.05% in the ARB+CCB group and 25.11% ± 12.67% in the ARB+diuretics group, respectively (*p* = 0.001). The mean RC was 70.56% in the ACEI+diuretics group, which was significantly higher than 66.33% in the ARB+diuretics group (*p* = 0.026). As for mean PWV, although it was lower in the ARB+diuretics group than in the ACEI+CCB, ACEI+diuretics, and ARB+CCB groups, the difference was not significant (Table 6).

### 3.6. Advanced Central Hemodynamic Parameters

As indicated in Table 7, the mean PR was 1600.85 ± 118.92 dyn∗s/cm^5^ in the ACEI+CCB group, and it was significantly lower (*p* = 0.001) than 1853.30 ± 236.01 dyn∗s/cm^5^ in the ACEI+diuretics group, while the PR of other groups was comparable. The mean CO was 5.09 ± 0.80 mL/min in the ARB+CCB group, which was significantly higher (*p* = 0.001) than that in the ACEI+diuretics group and the ARB+diuretics group (4.33 ± 0.63 mL/min and 4.60 ± 0.67 mL/min, respectively). The mean SV was 63.41 ± 07.61 mL in patients from the ACEI+diuretics group, which was significantly higher (*p* = 0.001) than that in patients from the ACEI+CCB and ARB+CCB groups (54.09 ± 04.93 mL and 55.54 ± 08.37 mL, respectively), while the SV of other groups was comparable. The mean CI was 3.00 ± 0.42 L/min∗L/m^2^ in the ARB+CCB group, and it was significantly higher (*p* = 0.001) than that in the ACEI+diuretics and ARB+diuretics groups (2.44 ± 0.40 L/min∗L/m^2^ and 2.68 ± 0.41 L/min∗L/m^2^, respectively).

### 3.7. Subgroup Analysis

The mean values of outcome measure in patients on perindopril and the rest of the treatment groups are summarized in Table 8. Patients who were taking perindopril had significantly lower CABP and PWV than those with other treatments (*p* < 0.05).

## 4. Discussion

Drug combinations involving RAAS inhibitors are commonly used treatment modality for managing patients with hypertension [22–24]. CABP is a better indicator of future cardiovascular events than brachial pressure [9, 10]. However, there is scarcity of evidence for the differential effect of various drug combinations on different CABP indices in an Indian population. In this study, our main objective was to evaluate the effect of different antihypertensive drug combinations on various CABP indices in Indian patients having hypertension and compare the results between different treatment groups. Our study showed that out of the four RAAS-based treatment groups, effective improvement of the CABP indices was observed in patients who were being treated with ACEI either in a two-drug combination or alone. This has also been indicated by the subgroup analysis that CABP indices were significantly improved with perindopril, which is a type of long-acting ACEI. The majority of patients were on ARB-based combinations (77.9%), followed by ACEI-based combination (20.1%) treatment. Moreover, the BP control rate reported in our study was 36.4%, which is consistent with the low rate (2.4% to 38%) reported for India [25].

Patients receiving combination antihypertensive therapy achieve lower BP levels correlating with a significant reduction in the risk of CV and cerebrovascular events [26–28]. Therefore, the majority of clinical practice guidelines recommend combinations of antihypertensive drugs for optimal management [29–31]. Conforming to these guidelines, all the patients received combination antihypertensives, with a majority receiving RAAS in our study. In a meta-analysis, RAAS inhibition resulted in a significant 5% reduction in all-cause mortality (HR: 0.95, 95% CI: 0.91–1.00, *p* = 0.032); however, the observed treatment benefit was entirely from the class of ACEIs, with a significant 10% reduction in all-cause mortality (HR: 0.90, 95% CI: 0.84–0.97, *p* = 0.004), while ARB treatment did not provide any mortality reduction (HR: 0.99, 95% CI: 0.94–1.04, *p* = 0.683) [22]. This difference in effects has been attributed to the different modes of action of ACEIs vs. ARBs primarily because of the pleiotropic effects and beneficial role of the bradykinin pathway with ACEIs [32–34]. Further, the guidelines also recommend preferring ACEIs over ARBs, suggesting that the ARBs should be used in patients with intolerance to ACEIs [35]. The majority of patients (77.9%) were receiving ARBs, and only 20.1% were given ACEIs that showed a wide gap in the clinical practice in India. There is a need to reassess the current prescription patterns to ensure that optimal treatment options are prescribed to patients.

The brachial systolic blood pressure (SBP) always remains higher than central aortic SBP due to pulse pressure (PP) amplification that also holds true when the effects of various antihypertensive classes are considered [36–38]. Moreover, we observed similar findings in our study that showed higher brachial BP compared to the corresponding CABP in all the four treatment groups. Further, both the brachial SBP and central SBP levels were lower in patients receiving ACEI-based combinations compared to those receiving ARB-based combinations. Our results contradict the results reported by Ruilope and Schaefer, showing better reduction in the central BP with ARB (olmesartan) than ACEI (perindopril) [39]. Nonetheless, our results should be interpreted cautiously since it was an observational study that did not have sufficient statistical power for such a comparison. As a result of a significant reduction in central BP, the vascular age and augmentation pressure also tended to be lower with ACEI-based combination treatment in our study.

Emerging evidence suggests that the central BP provides additional information regarding cardiovascular risk beyond the peripheral BP. Although our study did not include antihypertensive combination other than those based on RAAS, convincing evidence shows that there are important differences between the classes of antihypertensive drugs regarding their effects on BP amplification. The newer antihypertensive drugs (ACEIs and ARBs) as well as nitrates are more effective with regard to BP amplification than the older drugs (diuretics and BBs), and there is compelling evidence on the detrimental effect of BBs (mainly atenolol) on central BBs [13]. Collectively, the routine use of CABP could be an effective tool in the optimal management of hypertension.

The major limitation of our study was the cross-sectional design that did not allow observation of baseline and follow-up data. Other limitations include small number of patients, no biochemical investigations, and limited antihypertensive classes for comparison.

## 5. Conclusion

In summary, the majority of our patients were treated by ARB-based combination of antihypertensives. Despite this, the most central hemodynamic parameters including vascular age were better in patients treated with ACEI than in those treated with ARB. Our results indicate a gap between routine clinical practice and evidence-based guidelines in Indian settings and identify a need to reevaluate the current antihypertensive prescription strategy. Our study also suggests the use of CABP in routine practice to achieve the optimal management of hypertension.

## Figures and Tables

**Table 1:** Demographic characteristics of the analyzed population.

Total number of patients (*n* = 199)	
Gender, male	118 (59.3)
Age, 28-78 years	54.22 ± 10.15^∗^
Profile of controlled hypertension	72 (36.2)
Duration of hypertension	
≤3	62 (31.2)
>3	137 (67.8)
Profile of side effects	13 (6.5)
Risk factors (%)	
Family history of hypertension	138 (69.3)
Smoking	37 (18.6)
Tobacco chewing	30 (15.1)
Diabetes	11 (5.5)

*n*: frequency; %: percentage; ∗: mean ± SD.

**Table 2:** Drug treatment in the study population.

Combinations	Total number of patients (*n* = 199)
ACEI+CCB	13 (6.5)
ACEI+diuretics	27 (13.6)
ARB+CCB	65 (32.7)
ARB+diuretics	90 (45.2)
Others	4 (2.0)

*n*: frequency; ACEI: angiotensin-converting enzyme inhibitor; ARB: angiotensin II receptor blocker; CCB: calcium channel blocker.

**Table 3:** Peripheral blood pressure measurements among various drug combinations.

Treatment group	SBP (mmHg) mean ± SD	DBP (mmHg) mean ± SD	MAP (mmHg) mean ± SD	PP (mmHg) mean ± SD
ACEI+CCB	134.31 ± 11.35	80.77 ± 09.05	105.23 ± 08.87	53.54 ± 10.22
ACEI+diuretics	134.93 ± 21.96	88.04 ± 12.30	109.44 ± 15.52	46.89 ± 15.10
ARB+CCB	143.72^∗^ ± 18.62	90.78 ± 15.27^∗^	114.25 ± 14.51^∗^	52.51 ± 16.54
ARB+diuretics	141.69 ± 18.49	86.62 ± 11.65	111.63 ± 13.85	55.24 ± 13.58^∗^
Others	154.25 ± 22.69	104.50 ± 22.81	127.25 ± 22.74	49.75 ± 01.26
*p* value	0.027	0.009	0.014	0.031

*n*: frequency; ACEI: angiotensin-converting enzyme inhibitor; ARB: angiotensin II receptor blocker; CCB: calcium channel blocker; SBP: systolic blood pressure; DBP: diastolic blood pressure; MAP: mean arterial pressure; PP: pulse pressure. ^∗^Statistically significant; PP is defined as difference in mean SBP and DBP; MAP is defined as DBP + PP/3.

**Table 4:** Central blood pressure measurements.

Treatment group	SBP (mmHg) mean ± SD	DBP (mmHg) mean ± SD	PP (mmHg) mean ± SD	PPA mean ± SD	HR (1/m) mean ± SD
ACEI+CCB	119.92 ± 10.39	82.38 ± 09.33	37.23 ± 03.11	1.44 ± 0.21^∗^	88.46 ± 12.26
ACEI+diuretics	124.48 ± 19.46	89.11 ± 12.47	35.33 ± 10.12	1.30 ± 0.13	68.56 ± 05.52
ARB+CCB	132.69 ± 18.26^∗^	92.95 ± 15.33^∗^	39.57 ± 15.53	1.38 ± 0.20	92.63 ± 14.12^∗^
ARB+diuretics	127.63 ± 15.80	88.16 ± 11.63	39.42 ± 09.31	1.40 ± 0.16	78.11 ± 12.47
Others	143.75 ± 26.54	106.50 ± 23.17	37.25 ± 04.27	1.50 ± 0.32	90.00 ± 21.69
*p* value	0.001	0.001	—	0.001	0.006

*n*: frequency; ACEI: angiotensin-converting enzyme inhibitor; ARB: angiotensin II receptor blocker; CCB: calcium channel blocker; SBP: systolic blood pressure; DBP: diastolic blood pressure; PP: pulse pressure; PPA: pulse pressure amplification; m: meters. ^∗^Statistically significant; PP is defined as difference in mean SBP and DBP; PPA is defined as ([peripheral PP − central PP/central PP] × 100) by indirect derived calculations.

**Table 5:** Measurement of vascular age.

Mean actual age vs. vascular age (years)	
Actual age	54.22 ± 10.15
Vascular age	58.13 ± 12.43^∗^
Treatment group	
ACEI+CCB	60.85 ± 09.25
ACEI+diuretics	53.30 ± 13.38
ARB+CCB	60.55 ± 10.77^$^
ARB+diuretics	57.40 ± 13.32
Others	58.75 ± 13.20

*n*: frequency; ACEI: angiotensin-converting enzyme inhibitor; ARB: angiotensin II receptor blocker; CCB: calcium channel blocker. ^∗^*p* < 0.001; ^$^*p* = 0.034; vascular age (years) is the age of vessels older than biological age or the same as the biological age.

**Table 6:** Measurement of arterial stiffness.

Treatment group	AP (mmHg) mean ± SD	AI (%) mean ± SD	RC (%) mean ± SD	PWV (m/s) mean ± SD
ACEI+CCB	10.08 ± 06.29	32.38 ± 19.27	68.85 ± 07.06	9.03 ± 2.28
ACEI+diuretics	07.11 ± 04.67	17.67 ± 12.16	70.56 ± 08.45^∗^	8.50 ± 1.51
ARB+CCB	11.97 ± 09.71^∗^	34.82 ± 15.05^∗^	66.48 ± 10.31	8.66 ± 1.85
ARB+diuretics	10.60 ± 07.31	25.11 ± 12.67	66.33 ± 08.91	8.22 ± 1.67
Others	12.25 ± 05.12	41.00 ± 14.85	59.25 ± 13.99	7.98 ± 0.62
*p* value	0.001	0.001	0.026	—

*n*: frequency; ACEI: angiotensin-converting enzyme inhibitor; ARB: angiotensin II receptor blocker; CCB: calcium channel blocker; AP: augmentation pressure; AI: augmentation index; RC: reflection coefficient; PWV: pulse wave velocity; m: meters; s = seconds; ^∗^*p* = 0.001; ^#^*p* = 0.026.

**Table 7:** Advanced hemodynamic measurements.

Treatment group	PR (dyn∗s/cm^5^) mean ± SD	CO (L/min) mean ± SD	SV (mL) mean ± SD	CI (L/min∗L/m^2^) mean ± SD
ACEI+CCB	1600.85 ± 118.92	4.92 ± 0.77	54.09 ± 04.93	2.95 ± 0.52
ACEI+diuretics	1853.30 ± 236.01^∗^	4.33 ± 0.63	63.41 ± 07.61^∗^	2.44 ± 0.40
ARB+CCB	1718.88 ± 175.65	5.09 ± 0.80^∗^	55.54 ± 08.37	3.00 ± 0.42^∗^
ARB+diuretics	1777.50 ± 297.42	4.60 ± 0.67	59.28 ± 06.82	2.68 ± 0.41
Others	1881.50 ± 256.91	5.25 ± 1.68	57.32 ± 07.92	2.83 ± 0.76

*n*: frequency; ACEI: angiotensin-converting enzyme inhibitor; ARB: angiotensin II receptor blocker; CCB: calcium channel blocker; PR: peripheral resistance; CO: cardiac output; SV: stroke volume; CI: cardiac index; m: meters; dyn: dynes; ^∗^*p* = 0.001.

**Table 8:** Peripheral and central blood pressure indices in a subgroup of patients treated with perindopril vs. others.

	Perindopril (*n* = 22)	Others (*n* = 177)	*p* value
	Mean ± SD	Mean ± SD	
Peripheral blood pressure			
SBP (mmHg)	127.41 ± 9.65^∗^	142.92 ± 19.13	<0.001
DBP (mmHg)	83.00 ± 8.99^∗^	88.79 ± 13.81	0.012
MAP (mmHg)	103.2 7 ± 7.66	113.18 ± 14.73	—
PP (mmHg)	44.41 ± 10.53^∗^	54.06 ± 14.78	<0.001
Central blood pressure			
SBP (mmHg)	117.18 ± 9.79^∗^	130.11 ± 17.68	<0.001
DBP (mmHg)	84.00 ± 8.97^∗^	90.27 ± 13.89	0.005
PP (mmHg)	32.86 ± 5.33^∗^	39.46 ± 11.91	<0.001
PPA (mmHg)	1.33 ± 0.16	1.39 ± 0.18	—
HR (L/min)	73.95 ± 13.72^∗^	83.43 ± 14.90	0.005
Arterial stiffness			
AP (mmHg)	8.59 ± 5.50	10.82 ± 8.13	—
AI (%)	16.27 ± 17.76	28.28 ± 14.81	—
RC (%)	72.00 ± 7.47^∗^	66.35 ± 9.48	0.003
PWV (%)	9.25 ± 1.81^∗^	14.46 ± 20.28	0.001
Advanced hemodynamics			
PR (dyn∗s/cm^5^)	1760.1 ± 216.16	1759.1 ± 254.98	—
CO (L/min)	4.4 ± 0.679^∗^	4.8 ± 0.7955	0.02
SV (mL)	59.15 ± 6.50	58.12 ± 7.98	—
CI (L/min∗L/m^2^)	2.56 ± 0.415^∗^	2.79 ± 0.467	0.02
Vascular age (years)	56.13 ± 12.37	58.53 ± 12.34	—

SBP: systolic blood pressure; DBP: diastolic blood pressure; MAP: mean arterial pressure; PP: pulse pressure; PPA: pulse pressure amplification; AP: augmentation pressure; AI: augmentation index; RC: reflection coefficient; PWV: pulse wave velocity, PR: peripheral resistance; CO: cardiac output; SV: stroke volume; CI: cardiac index; m: meters; dyn: dynes; s: seconds. ^∗^Statistically significant; PP is defined as difference in mean SBP and DBP; MAP is defined as DBP + PP/3; PPA is defined as ([peripheral PP − central PP/central PP] × 100) by indirect derived calculations; vascular age (years) is the age of vessels older than biological age or the same as the biological age.

## Data Availability

The data used to support the findings of this study are available with the corresponding author and can be made available upon request.
